# Increased Uric Acid, Gamma-Glutamyl Transpeptidase and Alkaline Phosphatase in Early-Pregnancy Associated With the Development of Gestational Hypertension and Preeclampsia

**DOI:** 10.3389/fcvm.2021.756140

**Published:** 2021-10-15

**Authors:** Yequn Chen, Weichao Ou, Dong Lin, Mengyue Lin, Xiru Huang, Shuhua Ni, Shaoxing Chen, Jian Yong, Mary Clare O'Gara, Xuerui Tan, Ruisheng Liu

**Affiliations:** ^1^First Affiliated Hospital of Shantou University Medical College, Shantou, China; ^2^Shantou University Medical College, Shantou, China; ^3^Morsani College of Medicine, University of South Florida, Tampa, FL, United States

**Keywords:** biomarker, blood pressure, hypertensive disorders of pregnancy, longitudinal cohort study, pregnancy

## Abstract

**Background:** Previous studies have reported that biomarkers of liver injury and renal dysfunction were associated with hypertensive disorders of pregnancy (HDP). However, the associations of these biomarkers in early pregnancy with the risk of HDP and longitudinal blood pressure pattern during pregnancy were rarely investigated in prospective cohort studies.

**Methods:** A total of 1,041 pregnant women were enrolled in this prospective cohort study. BP was assessed in four stages throughout pregnancy. The following biomarkers were measured at early pregnancy before 18 weeks gestation: lactate dehydrogenase (LDH), aspartate aminotransferase to alanine aminotransferase ratio (AST/ALT), gamma-glutamyl transpeptidase (GGT), alkaline phosphatase (ALP), uric acid (UA), and estimated glomerular filtration rate (eGFR). Linear mixed-effects and logistic regression models were used to examine the associations of these biomarkers with longitudinal BP pattern during pregnancy and HDP incidence, respectively.

**Results:** In unadjusted models, higher serum UA, GGT, ALP, and LDH levels, as well as lower eGFR and AST/ALT, were associated with higher BP levels during pregnancy and an increased risk of HDP. After adjustment for maternal age, pre-pregnancy BMI and other potential confounders, UA, GGT, ALP, and LDH remained positively associated with both BP and HDP. However, eGFR and AST/ALT were not associated with HDP after adjusting for potential confounders. When including all 6 biomarkers simultaneously in multivariable analyses, increased UA, GGT, and ALP significantly associated with gestational hypertension and preeclampsia.

**Conclusion:** This study suggests that increased UA, GGT, and ALP in early-pregnancy are independent risk factors of gestational hypertension and preeclampsia.

## Introduction

Hypertensive disorders of pregnancy (HDP) affect up to 10% of pregnant women and remain major causes of maternal and perinatal morbidity and mortality worldwide (1–5). Identifying women at risk early in the course of such disorders, especially gestational hypertension and preeclampsia would facilitate intensive monitoring and intervention. Serum gamma-glutamyl transpeptidase (GGT), alkaline phosphatase (ALP), lactate dehydrogenase (LDH), aspartate aminotransferase to alanine aminotransferase ratio (AST/ALT) are common biomarkers of liver injury (6–8). Plasma creatinine (Cr), uric acid (UA), and estimated glomerular filtration rate (eGFR) are widely used as indicators for renal dysfunction (9, 10). Previous case-control studies have found that serum GGT, ALP, LDH, and UA levels are increased in women diagnosed with HDP (11–17). Recently, several studies have reported that lower AST/ALT was associated with insulin resistance, metabolic syndrome and cardiovascular disease (18–20). However, the associations of these biomarkers in early pregnancy with the risk of HDP were rarely investigated in prospective cohort studies.

Blood pressure (BP) changes progressively during pregnancy (21). In normal pregnancy, BP initially decreases until mid-pregnancy, and subsequently increases until delivery (21–23). A longitudinal study by Kac and colleagues recently reported that elevation of ALP, ALT, and Cr levels in the first trimester were associated with increased BP levels during pregnancy (24). As noted by the authors, the study involved a relatively small sample size and the study itself pertained to normotensive pregnant women. The associations reported prompt further investigation in large prospective cohort studies and the general population.

Accordingly, we investigated the associations of 6 hepatic and renal function biomarkers (GGT, ALP, AST/ALT, LDH, UA, and eGFR) in early pregnancy with longitudinal BP pattern during pregnancy and the risk of HDP in a large prospective cohort.

## Materials and Methods

### Study Population

This study was embedded in a population-based prospective cohort of pregnant women who received antenatal care in the First Affiliated Hospital of Shantou University Medical College in Shantou, China. Clinical characteristics of participants were assessed by questionnaire and physical examination at 7th−18th, 19th−27th, 28th−34th, and 35th−39th gestational weeks. Subjects were scheduled at enrollment for clinic visits during follow-up. Patients who missed their scheduled visits would be contacted for clinic visits, whereupon the patient's information would be collected. For patients who could not attend clinical visits, patients' information would be collected by telephone interviews with their families and electronic medical records. If patients could not be followed up through in-clinic visits, telephone interviews, or electronic medical records, such patients were recorded as a loss to follow-up. A total of 2,206 pregnant women were accumulatively recruited from March 2014 to April 2016 inclusive. Included were women who had baseline measurement of hepatic and renal biochemical function before 18 weeks gestation (*N* = 1,371). Exclusion criteria included *in vitro* fertilization or twin pregnancies (*N* = 16), miscarriage (*N* = 9), loss to follow-up (*N* = 221), or having been diagnosed with chronic hepatitis B (*N* = 74), chronic nephritis (*N* = 5), rheumatoid arthritis (*N* = 3), or systemic lupus erythematosus (*N* = 2). After exclusion, 1,041 participants were enrolled.

### Blood Pressure Measurements

BP was assessed in four stages during pregnancy (7th−18th, 19th−27th, 28th−34th, and 35th−39th gestational weeks). At each visit, BP was measured thrice in the morning by qualified nurses using an Omron HEM-7052 automatic blood pressure monitor (Omron Healthcare Ltd., Dalian, China) according to the standard measurement procedure recommended by the American Heart Association (25). The mean of 3 readings was used in the analysis. Some subjects had missing BP measurements due to missed visits. In total, 3,274 blood pressure measurements were available for analysis, of which 31 subjects had one measurement, 225 subjects had two measurements, 347 subjects had 3 measurements, and 438 subjects had 4 measurements.

### Hypertensive Disorders of Pregnancy

Maternal outcomes were obtained from medical records. A total of 60 pregnant women met the criteria of HDP, including 7 with chronic hypertension, 22 with gestational hypertension, and 31 experienced preeclampsia.

Chronic hypertension was defined as SBP ≥ 140 mmHg and/or DBP ≥ 90 mmHg before 20 weeks gestation. Gestational hypertension was defined as SBP ≥ 140 mmHg and/or DBP ≥ 90 mmHg without proteinuria, which had developed for the first time after 20 weeks gestation (26). Preeclampsia was defined as SBP ≥ 140 mmHg and/or DBP ≥ 90 mmHg with proteinuria (defined as ≥300 mg of protein in a 24-h urine specimen or ≥1+ in two random urine samples collected at least 4 h apart) (26).

### Laboratory Analysis

Hepatic and renal function biochemical testing (comprising LDH, AST, ALT, ALP, GGT, UA, and Cr) was performed in the Department of Clinical Laboratory of the First Affiliated Hospital of Shantou University Medical College, using an automatic biochemical analyzer (Beckman counter AU5800, USA). Maternal overnight fasting blood samples were collected in the morning during early pregnancy (median 13.4 weeks, 95% range 7.1–18.0). The eGFR was calculated using the Chronic Kidney Disease Epidemiology Collaboration (CKD-EPI) formula (27).

### Covariates

Maternal age, monthly per capita income, education level, nulliparous (yes or no), folic acid supplement intake during pregnancy (yes or no), family history of hypertension (yes or no), family history of diabetes mellitus (yes or no), smoking habit (yes or no), alcohol consumption (yes or no), pre-pregnancy weight and height were obtained by questionnaire on enrolment. Pre-pregnancy BMI was calculated as pre-pregnancy weight/height^2^ (kg/m^2^) (28).

### Statistical Analysis

The hepatic and renal function biomarkers were categorized into tertiles and were analyzed as categorical variables (24). Linear mixed-effects regression models were used to evaluate the association of biomarkers with SBP and DBP change during pregnancy (22). This regression technique considers the correlation of repeated measurements in the same individual and allows incomplete outcome data, commonly applied to the analysis of repeated measurement data (29). Gestational age (linear and quadratic terms) was included in the linear mixed-effects regression models to fit the quadratic function of the association of blood pressure with time (24). Gestational age was included in the models as both random and fixed effects, whereas biomarkers and other covariates were analyzed as fixed effects. Maternal age, BMI, income, education, folic acid supplementation, family history of hypertension, family history of diabetes mellitus, as well as smoking and alcohol consumption have been reported to be associated with HDP (30–32). In order to adjust for these potential confounders, we applied 3 models: Model 1 was adjusted for gestational age (linear and quadratic terms); Model 2 was further adjusted for pre-pregnancy BMI, maternal age, monthly per capita income, education level, parity, folic acid supplementation during pregnancy, family history of hypertension, family history of diabetes mellitus, and smoking and alcohol consumption; Model 3 included all six biomarkers simultaneously and was adjusted for covariates as in Model 2. To further assess whether similar associations were also present in a normotensive population, we repeated the analyses in women who remained normotensive throughout pregnancy. The curve of longitudinal blood pressure change with gestational age was estimated using a crude linear mixed-effects regression model.

The associations of 6 early-pregnancy biomarkers with the risk of HDP were analyzed using three logistic regression models: Model 1 was unadjusted; Model 2 was adjusted for pre-pregnancy BMI, maternal age, monthly per capita income, education level, parity, folic acid supplement intake during pregnancy, family history of hypertension, family history of diabetes mellitus, smoking, alcohol consumption, and gestational age at sampling; Model 3 included all six biomarkers simultaneously and was adjusted for covariates as in Model 2.

In addition, among normal pregnancy, gestation hypertension, and preeclampsia, one way ANOVA was performed to analyze the difference of maternal age, pre-pregnancy BMI, gestational age at sampling weeks, UA, eGFR, GGT, ALP, and LDH. Chi-square was used to analyze the difference of monthly per capita income, education level, nulliparous, folic acid supplement intake during pregnancy, family history of hypertension, family history of diabetes mellitus, smoking habit, and alcohol consumption.

All statistical analyses were performed using SPSS version 19.0 (SPSS Inc., Chicago, Illinois, USA). Two-tailed *P*-values < 0.05 were considered statistically significant.

## Results

### Participant Characteristics

Baseline characteristics of the study population are shown in Table 1. Mean maternal age and pre-pregnancy BMI were 29.5 ± 4.3 years and 20.5 ± 2.9 kg/m^2^, respectively. Of all women included in the study, 44.3% was nulliparous and 19.9% had a family history of hypertension. At follow-up, 60 (5.8%) pregnant women had developed HDP.

**Table 1:** Baseline characteristics of the study population.

**Characteristics**	**Total (*N* = 1,041)**
Age, y	29.5 ± 4.3
Pre-pregnancy BMI, kg/m^2^	20.5 ± 2.9
**Monthly per capita income**	
<3,000 RMB	392 (37.7)
3,000–5,000 RMB	355 (34.1)
>5,000 RMB	294 (28.2)
**Education level**	
Below high school	226 (21.7)
High school	223 (21.4)
Beyond high school	592 (56.9)
Nulliparous	461 (44.3)
Folic acid supplement	761 (73.1)
Family history of hypertension	208 (19.9)
Family history of diabetes mellitus	138 (13.3)
Smoking	25 (2.4)
Alcohol consumption	300 (28.8)
HDP	60 (5.8)
Gestational age at sampling, weeks	13.7 ± 3.1
UA, μmol/L	241.8 ± 50.1
eGFR, mL/min/1.73 m^2^	112.2 ± 10.8
GGT, U/L	15.1 ± 8.8
ALP, U/L	49.7 ± 13.7
LDH, U/L	150.8 ± 21.9
AST/ALT	1.19 ± 0.42

*Data are presented as means ± standard deviation or n (%). BMI, body mass index; HDP, hypertensive disorders of pregnancy; UA, uric acid; eGFR, estimated glomerular filtration rate; GGT, gamma–glutamyl transpeptidase; ALP, alkaline phosphatase; LDH, lactate dehydrogenase; AST/ALT, aspartate aminotransferase to alanine aminotransferase ratio*.

### Serum Biomarkers and Blood Pressure Pattern During Pregnancy

**Figure 1:**
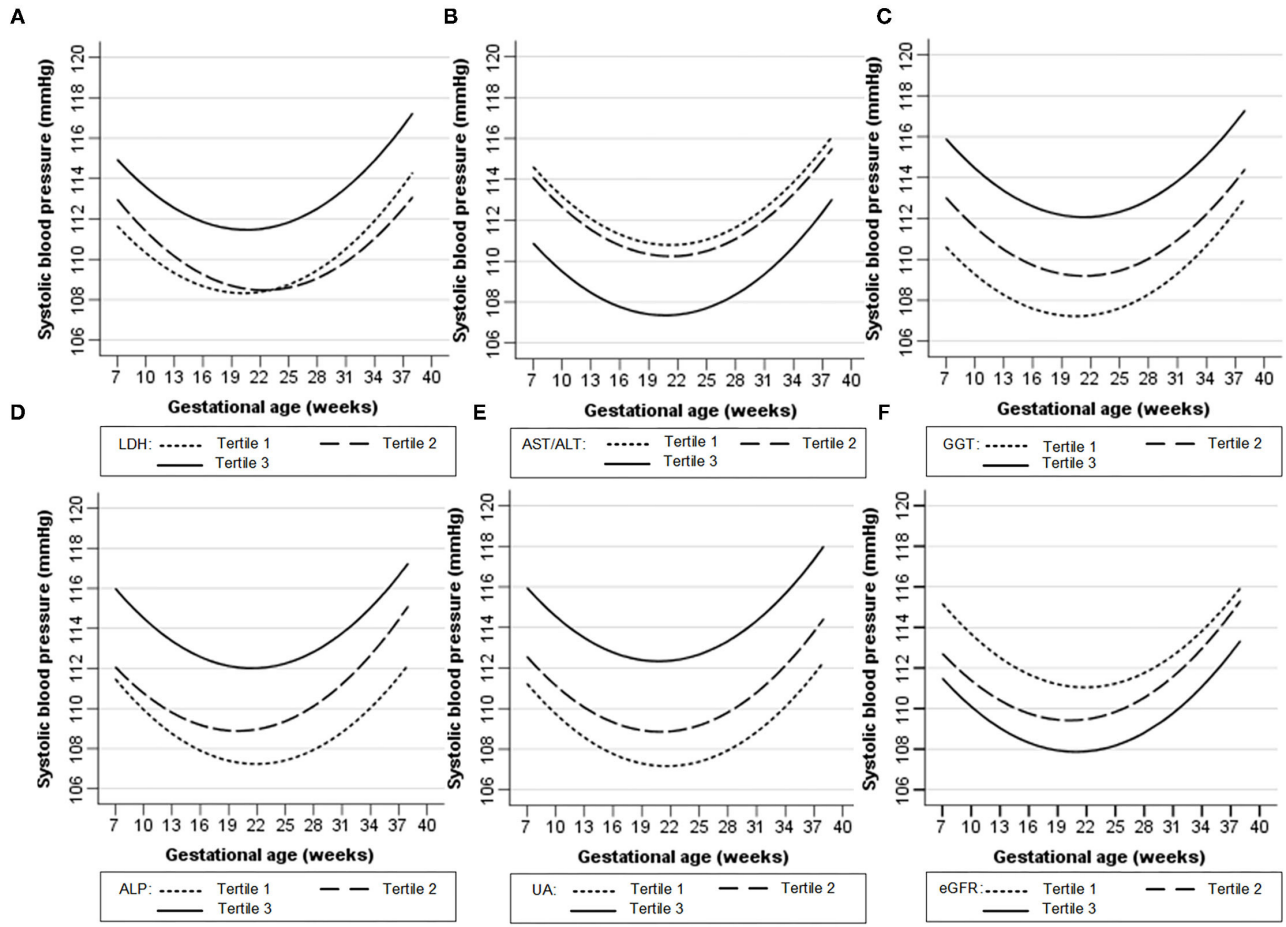
Longitudinal trend of systolic blood pressure change with gestational age vs. early-pregnancy biomarkers levels, predicted by linear mixed-effects regression models. **(A)** lactate dehydrogenase (LDH); **(B)** aspartate aminotransferase to alanine aminotransferase ratio (AST/ALT); **(C)** gamma-glutamyl transpeptidase (GGT); **(D)** alkaline phosphatase (ALP); **(E)** uric acid (UA); **(F)** estimated glomerular filtration rate (eGFR).

**Figure 2:**
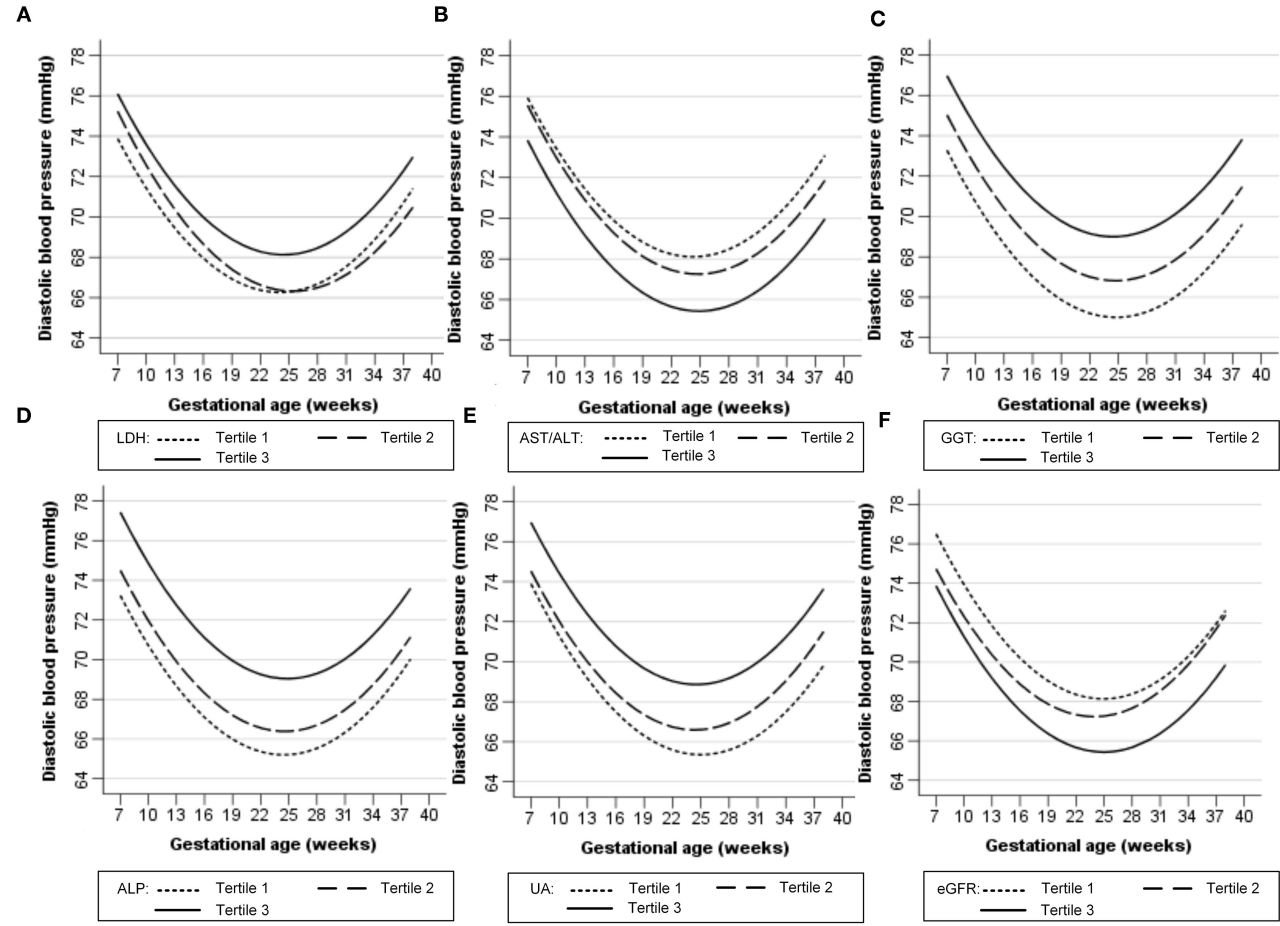
Longitudinal trend of diastolic blood pressure change with gestational age vs. early-pregnancy biomarkers levels, predicted by linear mixed-effects regression models. **(A)** lactate dehydrogenase (LDH); **(B)** aspartate aminotransferase to alanine aminotransferase ratio (AST/ALT); **(C)** gamma-glutamyl transpeptidase (GGT); **(D)** alkaline phosphatase (ALP); **(E)** uric acid (UA); **(F)** estimated glomerular filtration rate (eGFR).

Figures 1, 2 show the longitudinal patterns of SBP and DBP change and early pregnancy biomarker level during different tertiles of pregnancy. The distribution of maternal blood pressure throughout pregnancy was shown in Supplementary Figures S1, S2. Women in the third tertile of LDH, GGT, ALP, and UA levels had higher SBP and DBP than those in the first tertile, whereas women in the third tertile of AST/ALT and eGFR had lower SBP and DBP during pregnancy than those in the first tertile.

Tables 2, 3 show the longitudinal associations of early-pregnancy biomarkers with SBP and DBP levels during pregnancy, respectively. In crude analyses (Model 1), higher UA, GGT, ALP, and LDH levels, as well as lower eGFR and AST/ALT, were associated with higher SBP and DBP levels during pregnancy. After adjustment for maternal age, pre-pregnancy BMI and other potential confounders (Model 2), higher UA, GGT, ALP, LDH, and AST/ALT remained associated with both increased SBP and DBP, whereas lower eGFR was only associated with increased DBP. When all 6 biomarkers were simultaneously included in multivariable analyses (Model 3), only increased UA, GGT, and ALP remained associated with increased BP. Similar results were found in pregnant women without HDP (Supplementary Tables S1, S2).

**Table 2:** Longitudinal associations of early-pregnancy biomarkers levels with systolic blood pressure levels during pregnancy.

**Biomarkers**	***N***	Model 1^*^	**Model 2^**†**^**	**Model 3^**‡**^**
**UA**, **μmol/L**				
T1 (125–218)	349	Reference	Reference	Reference
T2 (219–256)	347	1.81 (0.31, 3.31)^§^	1.54 (0.06, 3.03)^§^	1.22 (-0.27, 2.71)
T3 (257–430)	345	5.29 (3.78, 6.80)^∥^	3.75 (2.24, 5.27)^∥^	3.15 (1.63, 4.68)^∥^
**eGFR, mL/min/1.73 m** ^**2**^				
T1 (68–109)	366	3.07 (1.5, 4.64)^∥^	0.87 (−0.75, 2.49)	0.25 (−1.35, 1.86)
T2 (110–118)	321	1.65 (0.02, 3.28)^§^	0.29 (−1.31, 1.89)	−0.08 (−1.66, 1.51)
T3 (119–137)	354	Reference	Reference	Reference
**GGT, U/L**				
T1 (4–10)	336	Reference	Reference	Reference
T2 (11–15)	375	1.85 (0.27, 3.42)^§^	1.15 (−0.35, 2.66)	0.69 (−0.84, 2.22)
T3 (16–68)	330	4.72 (3.10, 6.34)^∥^	3.11 (1.56, 4.67)^∥^	2.04 (0.31, 3.76)^§^
**ALP, U/L**				
T1 (18–43)	341	Reference	Reference	Reference
T2 (44–52)	332	1.92 (0.31, 3.53)^§^	1.57 (0.06, 3.08)^§^	1.16 (−0.34, 2.67)
T3 (53–193)	368	4.83 (3.26, 6.40)^∥^	3.2 (1.69, 4.71)^∥^	2.32 (0.78, 3.86)^∥^
**LDH, U/L**				
T1 (97–139)	333	Reference	Reference	Reference
T2 (140–157)	353	−0.13 (−1.74, 1.48)	−0.02 (−1.52, 1.48)	−0.38 (−1.87, 1.10)
T3 (158–273)	355	3.08 (1.47, 4.69)^∥^	2.44 (0.94, 3.94)^∥^	1.48 (−0.08, 3.27)
**AST/ALT**				
T1 (0.38–0.97)	340	3.36 (1.75, 4.97)^∥^	1.68 (0.11, 3.25)^§^	0.09 (−1.62, 1.79)
T2 (1.00–1.29)	355	2.80 (1.21, 4.39)^∥^	1.49 (−0.03, 3.00)	0.76 (−0.78, 2.30)
T3 (1.30–3.40)	346	Reference	Reference	Reference

*Results were regression coefficients (95% confidence interval) of linear mixed-effects regression models. UA, uric acid; eGFR, estimated glomerular filtration rate; GGT, gamma–glutamyl transpeptidase; ALP, alkaline phosphatase; LDH, lactate dehydrogenase; AST/ALT, aspartate aminotransferase to alanine aminotransferase ratio; T, tertile*.

* *Model 1 was adjusted for gestational age (linear and quadratic terms)*.

† *Model 2 was adjusted for gestational age (linear and quadratic terms), pre-pregnancy BMI, maternal age, monthly per capita income, education level, nulliparous, folic acid supplement intake during pregnancy, family history of hypertension, family history of diabetes mellitus, smoking and alcohol consumption*.

‡ *Model 3 included all six biomarkers simultaneously and was adjusted for gestational age (linear and quadratic terms), pre-pregnancy BMI, maternal age, monthly per capita income, education level, nulliparous, folic acid supplement intake during pregnancy, family history of hypertension, family history of diabetes mellitus, smoking and alcohol consumption*.

§ *P < 0.05*.

∥ *P < 0.01*.

**Table 3:** Longitudinal associations of early-pregnancy biomarkers levels with diastolic blood pressure levels during pregnancy.

**Biomarkers**	***N***	Model 1^*^	**Model 2^**†**^**	**Model 3^**‡**^**
**UA**, **μmol/L**				
T1 (125–218)	349	Reference	Reference	Reference
T2 (219–256)	347	1.27 (0.07, 2.47)^§^	1.12 (−0.01, 2.25)	0.96 (−0.17, 2.09)
T3 (257–430)	345	3.52 (2.32, 4.73)^∥^	2.37 (1.21, 3.52)^∥^	1.88 (0.73, 3.04)^∥^
**eGFR, mL/min/1.73 m** ^**2**^				
T1 (68–109)	366	2.70 (1.51, 3.88)^∥^	1.28 (0.09, 2.46)^§^	0.88 (−0.33, 2.10)
T2 (110–118)	321	1.85 (0.62, 3.08)^∥^	1.02 (−0.15, 2.19)	0.73 (−0.47, 1.93)
T3 (119–137)	354	Reference	Reference	Reference
**GGT, U/L**				
T1 (4–10)	336	Reference	Reference	Reference
T2 (11–15)	375	1.82 (0.64, 3.01)^∥^	1.27 (0.13, 2.40)^§^	0.99 (−0.17, 2.15)
T3 (16–68)	330	4.01 (2.79, 5.23)^∥^	2.80 (1.63, 3.98)^∥^	1.93 (0.63, 3.24)^∥^
**ALP, U/L**				
T1 (18–43)	341	Reference	Reference	Reference
T2 (44–52)	332	1.18 (−0.04, 2.39)	0.95 (−0.20, 2.09)	0.62 (−0.52, 1.76)
T3 (53–193)	368	3.82 (2.64, 5.01)^∥^	2.62 (1.47, 3.76)^∥^	1.98 (0.81, 3.14)^∥^
**LDH, U/L**				
T1 (97–139)	333	Reference	Reference	Reference
T2 (140–157)	353	0.01 (−1.22, 1.22)	0.12 (−1.02, 1.26)	−0.18 (−1.3, 0.95)
T3 (158–273)	355	1.83 (0.61, 3.05)^∥^	1.37 (0.23, 2.51)^§^	0.76 (−0.38, 1.90)
**AST/ALT**				
T1 (0.38–0.97)	340	2.70 (1.48, 3.92)^∥^	1.45 (0.26, 2.64)^§^	0.18 (−1.11, 1.47)
T2 (1.00–1.29)	355	1.82 (0.62, 3.02)^∥^	0.87 (−0.28, 2.02)	0.25 (−0.91, 1.42)
T3 (1.30–3.40)	346	Reference	Reference	Reference

*Results were regression coefficients (95% confidence interval) of linear mixed-effects regression models. UA, uric acid; eGFR, estimated glomerular filtration rate; GGT, gamma–glutamyl transpeptidase; ALP, alkaline phosphatase; LDH, lactate dehydrogenase; AST/ALT, aspartate aminotransferase to alanine aminotransferase ratio; T, tertile*.

* *Model 1 was adjusted for gestational age (linear and quadratic terms)*.

† *>Model 2 was adjusted for gestational age (linear and quadratic terms), pre-pregnancy BMI, maternal age, monthly per capita income, education level, nulliparous, folic acid supplement intake during pregnancy, family history of hypertension, family history of diabetes mellitus, smoking and alcohol consumption*.

‡ *Model 3 included all six biomarkers simultaneously and was adjusted for gestational age (linear and quadratic terms), pre-pregnancy BMI, maternal age, monthly per capita income, education level, nulliparous, folic acid supplement intake during pregnancy, family history of hypertension, family history of diabetes mellitus, smoking and alcohol consumption*.

§ *P < 0.05*.

∥ *P < 0.01*.

### Serum Biomarkers and HDP

Table 4 presents the association of early-pregnancy biomarkers with the risk of HDP, using multivariable logistic regression models. In unadjusted analyses (Model 1), higher UA, GGT, ALP, and LDH levels, as well as lower eGFR and AST/ALT, were associated with increased risk of HDP. After adjustment for maternal age, pre-pregnancy BMI and other potential confounders (Model 2), only increased UA, GGT, ALP, and LDH remained associated with the risk of HDP. When all 6 biomarkers were simultaneously included in multivariable analyses (Model 3), the highest tertile of UA, GGT, and ALP were associated with a higher risk of HDP (odds ratio, 3.57 [95% confidence interval, 1.36–9.39]; odds ratio, 2.61 [95% confidence interval, 1.05–6.83]; odds ratio, 2.12 [95% confidence interval, 1.01–4.90], respectively). The associations of LDH, eGFR and AST/ALT with HDP were no longer statistically significant following multivariate adjustment for UA, GGT, and ALP (Model 3).

**Table 4:** Associations of early-pregnancy biomarkers levels with risk of hypertensive disorders of pregnancy.

**Biomarkers**	***N***	Model 1^*^	**Model 2^**†**^**	**Model 3^**‡**^**
**UA**, **μmol/L**				
T1 (125–218)	349	Reference	Reference	Reference
T2 (219–256)	347	3.50 (1.39, 8.82)^∥^	3.25 (1.24, 8.53)^§^	2.91 (1.09, 7.82)^§^
T3 (257–430)	345	6.25 (2.59, 15.09)^∥^	4.48 (1.76, 11.41)^∥^	3.57 (1.36, 9.39)^∥^
**eGFR, mL/min/1.73 m** ^**2**^				
T1 (68–109)	366	3.09 (1.54, 6.22)^∥^	1.56 (0.67, 3.63)	1.14 (0.48, 2.75)
T2 (110–118)	321	1.64 (0.75, 3.58)	0.97 (0.40, 2.33)	0.75 (0.3, 1.87)
T3 (119–137)	354	Reference	Reference	Reference
**GGT, U/L**				
T1 (4–10)	336	Reference	Reference	Reference
T2 (11–15)	375	1.83 (0.77, 4.33)	1.29 (0.52, 3.19)	1.17 (0.45, 3.05)
T3 (16–68)	330	5.02 (2.3, 10.97)^∥^	3.39 (1.46, 7.85)^∥^	2.61 (1.05, 6.83)^§^
**ALP, U/L**				
T1 (18–43)	341	Reference	Reference	Reference
T2 (44–52)	332	1.75 (0.75, 4.05)	1.42 (0.58, 3.46)	1.13 (0.45, 2.87)
T3 (53–193)	368	4.00 (1.9, 8.44)^∥^	2.83 (1.27, 6.3)^§^	2.12 (1.01, 4.90)^§^
**LDH, U/L**				
T1 (97–139)	333	Reference	Reference	Reference
T2 (140–157)	353	1.17 (0.55, 2.47)	1.3 (0.59, 2.88)	1.14 (0.49, 2.63)
T3 (158–273)	355	2.36 (1.21, 4.58)^§^	2.1 (1.02, 4.3)^§^	1.66 (0.78, 3.54)
**AST/ALT**				
T1 (0.38–0.97)	340	2.40 (1.20, 4.82)^§^	1.71 (0.79, 3.68)	0.93 (0.38, 2.24)
T2 (1.00–1.29)	355	1.75 (0.85, 3.61)	1.15 (0.52, 2.52)	0.84 (0.36, 1.96)
T3 (1.30–3.40)	346	Reference	Reference	Reference

*Results were odds ratio (95% confidence interval) derived from logistic regression models. UA, uric acid; eGFR, estimated glomerular filtration rate; GGT, gamma–glutamyl transpeptidase; ALP, alkaline phosphatase; LDH, lactate dehydrogenase; AST/ALT, aspartate aminotransferase to alanine aminotransferase ratio; T, tertile*.

* *Model 1 was unadjusted*.

† *Model 2 was adjusted for pre-pregnancy BMI, maternal age, monthly per capita income, education level, nulliparous, folic acid supplement intake during pregnancy, family history of hypertension, family history of diabetes mellitus, smoking, alcohol consumption and gestational age at sampling*.

‡ *Model 3 including all six biomarkers simultaneously and was adjusted for pre-pregnancy BMI, maternal age, monthly per capita income, education level, nulliparous, folic acid supplement intake during pregnancy, family history of hypertension, family history of diabetes mellitus, smoking, alcohol consumption and gestational age at sampling*.

§ *P < 0.05*.

∥ *P < 0.01*.

### UA, GGT, and ALP Levels in Normal Pregnancy and HDP

**Figure 3:**
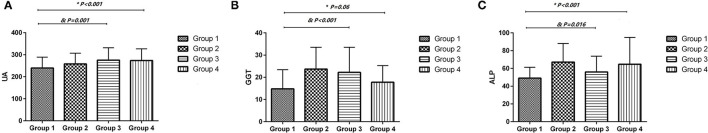
The uric acid, gamma-glutamyl transpeptidase and alkaline phosphatase levels were compared in normal pregnancy (group 1), gestation hypertension (group 3), and preeclampsia (group 4) by One way ANOVA. **(A)** uric acid (UA); **(B)** gamma-glutamyl transpeptidase (GGT); **(C)** alkaline phosphatase (ALP). Chronic hypertension (group 2) was not included for analysis due to small sample size. *Preeclampsia (group 4) compare to normal pregnancy (group1) by One way ANOVA. ^&^Gestation hypertension (group 3) compare to normal pregnancy (group1) by One way ANOVA.

Table 5 presents baseline characteristics of normal pregnancy (group 1), chronic hypertension (group 2), gestation hypertension (group 3), and preeclampsia (group 4). Since the sample size of chronic hypertension is too small, statistical analysis was only performed in groups 1, 3, and 4 and presented in Figure 3. UA and ALP levels were significantly higher in gestational hypertension and preeclampsia as compared to normal pregnancy.

**Table 5:** Baseline characteristics of normal pregnancy (group 1), chronic hypertension (group 2), gestation hypertension (group 3), and preeclampsia (group 4).

**Characteristics**	**Group 1**	**Group 2**	**Group 3**	**Group 4**	*P*-value^**^
	**(*n* = 981)**	**(*n* = 7)**	**(*n* = 22)**	**(*n* = 31)**	
Age, *y*	29.33 ± 4.20	35.86 ± 4.22	32.86 ± 5.66	32.06 ± 4.79	0.000^*^
Pre-pregnancy BMI, kg/m^2^	20.33 ± 2.76	25.33 ± 2.76	23.51 ± 3.59	22.15 ± 4.67	0.000^*^
Monthly per capita income					0.702^&^
<3,000 RMB	283 (28.8%)	–	4 (18.2%)	7 (22.6%)	
3,000–5,000 RMB	335 (34.1%)	1 (14.3%)	9 (40.9%)	10 (32.3%)	
>5,000 RMB	363 (37.0%)	6 (85.7%)	9 (40.9%)	14 (45.2%)	
Education level					0.378^&^
Below high school	565 (57.6%)	3 (42.9%)	10 (45.5%)	14 (45.2%)	
High school	209 (21.3%)		7 (31.8%)	7 (22.6%)	
Beyond high school	207 (21.1%)	4 (57.1%)	5 (22.7%)	10 (32.2%)	
Nulliparous	442 (45.1%)	1 (14.3%)	6 (27.3%)	12 (38.7%)	0.203^&^
Folic acid supplement	726 (74.0%)	5 (71.4%)	15 (68.2%)	15 (48.4%)	0.006^&^
Family history of hypertension	185 (18.9%)	5 (71.4%)	7 (31.8%)	11 (35.5%)	0.025^&^
Family history of diabetes mellitus	125 (12.7%)	1 (14.3%)	5 (22.7%)	7 (22.6%)	0.117^&^
Smoking	20 (2.0%)	–	2 (9.1%)	3 (9.7%)	0.003^&^
Alcohol consumption	287 (29.3%)	–	4 (18.2%)	9 (29.0%)	0.527^&^
Gestational age at sampling, weeks	13.67 ± 3.04	12.39 ± 3.92	13.88 ± 3.57	14.70 ± 3.01	0.172^*^
eGFR, mL/min/1.73 m^2^	112.59 ± 10.46	105.71 ± 7.84	104.11 ± 11.57	106.06 ± 15.87	0.000^*^
UA, μmol/L	239.96 ± 49.34	258.43 ± 48.86	275.43 ± 56.03	273.81 ± 52.54	0.000^*^
GGT, U/L	14.80 ± 8.63	23.71 ± 9.79	22.23 ± 11.28	17.77 ± 7.67	0.000^*^
ALP, U/L	49.02 ± 12.29	67.14 ± 21.10	55.95 ± 17.73	64.68 ± 30.19	0.000^*^
LDH, U/L	150.03 ± 21.11	162.57 ± 25.11	166.73 ± 30.31	159.90 ± 31.13	0.000^*^
AST/ALT	1.19 ± 0.42	0.86 ± 0.30	1.06 ± 0.27	1.10 ± 0.42	0.147^*^

*Data are presented as means ± standard deviation or n (%). BMI, body mass index; UA, uric acid; eGFR, estimated glomerular filtration rate; GGT, gamma–glutamyl transpeptidase; ALP, alkaline phosphatase; LDH, lactate dehydrogenase; AST/ALT, aspartate aminotransferase to alanine aminotransferase ratio*.

* *Analysis with one way ANOVA*,

& *Analysis with Chi-square*.

** *All statistical analysis was not included group 2 due to small sample size*.

## Discussion

In this population-based prospective cohort study, we demonstrate that higher serum UA, GGT, ALP, and LDH levels in early pregnancy, as well as lower eGFR and AST/ALT, are associated with higher BP levels during pregnancy and an increased risk of HDP. When including all 6 biomarkers simultaneously in multivariable analyses adjusted for potential confounders, increased levels of UA, GGT, and ALP were significantly associated with gestational hypertension and preeclampsia. Our study suggests that elevated UA, GGT, and ALP in early pregnancy are independent risk factors for the development of gestational hypertension and preeclampsia.

### UA

For UA, our results are consistent with previous cross-sectional and prospective studies demonstrating the positive association between UA and HDP (15, 33, 34). Furthermore, we find that increased serum UA in early pregnancy is independent factor associated with higher longitudinal BP progression during pregnancy. In contrast to our findings, Kac et al. reported UA in the first trimester was not associated with BP levels during pregnancy following adjustment for maternal BMI (24). This inconsistency may be due to differences in sample size and participant characteristics. The study, as stated by the authors, investigated a relatively small sample size of 225 and specifically studied normotensive pregnant women (24). However, when we repeated the analysis in pregnant women without HDP, increased UA remained associated with both higher SBP and DBP levels during pregnancy following adjustment for maternal BMI and other confounders (Supplementary Tables S1, S2).

Many experimental studies suggest that UA may play a casual role in the development of hypertension (14, 15). Hyperuricemia induced hypertension, which can be prevented by UA-lowering treatment (35). Hyperuricemia has been shown to stimulate the renin-angiotensin system, inhibit neuronal nitric oxide synthase and induce endothelial dysfunction (35, 36). In addition, UA has been reported to induce trophoblastic production of pro-inflammatory interleukin-1β through activation of inflammatory pathways (37). These underlying mechanisms may partly explain the role of UA in BP progression and the development of HDP.

### GGT

Serum GGT is a known biomarker for liver injury and alcohol consumption (38). Several longitudinal studies have reported GGT as positively associated with BP progression and the risk of hypertension in non- pregnant persons (39–41). A study of almost 12,000 hypertensive adults found that higher baseline GGT levels were associated with higher follow-up BP and an increased risk of cardiovascular mortality (42). However, no prior longitudinal study has investigated the associations of serum GGT in early pregnancy with longitudinal BP during pregnancy and the risk of HDP. In this present study, serum GGT in early pregnancy is positively associated with BP levels during pregnancy and a risk for HDP having adjusted for various confounders. Although exact mechanisms that link GGT with BP progression and HDP are not fully elucidated, several possible explanations are posited: GGT plays a role in the generation of free radical species through its interaction with iron and other transition metals (43). Serum GGT has been positively associated with inflammatory markers such as fibrinogen, C-reactive protein (CRP), and F2-isoprostanes. Thus, elevated GGT could potentially act as an additional marker for oxidative stress and inflammation, which are, as demonstrated by Palei et al., important features of HDP (44).

### ALP

In the current study, higher serum ALP in early pregnancy is also independently associated with elevated BP during pregnancy and increased HDP incidence. This finding confirms and extends the result of a previous study that has reported a positive association of ALP with BP during normal pregnancy (24). The relationship of ALP with BP and HDP may be partly explained by its correlation with vascular calcification. ALP is a hydrolase enzyme that catalyzes the hydrolysis of inorganic pyrophosphate, an inhibitor of vascular calcification (45). Increased levels of ALP have been found in vessels with medial calcification (46), and have been positively associated with higher risk of hypertension, peripheral arterial disease and cardiovascular diseases (47–49). In addition, many studies have reported that serum ALP positively correlated with the inflammatory marker, CRP (20, 50–52). High serum ALP levels may partially reflect the inflammatory process, which has been associated with the pathology of HDP (44). We further compared the levels of UA, GGT, and ALP between normal pregnancy and gestational hypertension and preeclampsia. UA, GGT, and ALP were associated preeclampsia (Figure 3).

In conclusion, our study provided evidence that higher UA, GGT, and ALP levels in early pregnancy are independent risk factors of gestational hypertension and preeclampsia. These findings suggest that UA, GGT, and ALP could be markers for the development of gestational hypertension and preeclampsia. However, it is not clear the elevations of UA, GGT, and ALP in early pregnancy is a casual factor or consequence of the development of gestational hypertension and preeclampsia. This would be an open and essential question for future studies that could provide new targets for treatment of gestational hypertension and preeclampsia. Our findings warrant further both clinical and experimental studies to identify the underlying mechanisms and clinical value in the early diagnosis, prevention and management of HDP.

### Study Strengths and Limitations

The strengths of our study include the large prospective population-based cohort from early pregnancy onwards, standardized measurement of BP, the ability to adjust for multiple traditional confounders and the ability to simultaneously assess multiple biomarkers. However, our study has some limitations. First, this was a single-center study with a small number of HDP events. Second, the serum concentrations of hepatic and renal biomarkers were assayed solely in early pregnancy. Thus, the correlation of blood pressure and HDP with these biomarkers measured before pregnancy and after delivery cannot be evaluated. Third, since BP varies during the day according to a circadian rhythm (53) and our study did not include ambulatory blood pressure measurements, this may introduce some random measurement error in the analysis. Finally, the sample size of chronic hypertension in pregnancy was too small to perform statistical analysis.

## Data Availability Statement

The raw data supporting the conclusions of this article will be made available by the authors, without undue reservation.

## Ethics Statement

The studies involving human participants were reviewed and approved by Research and Ethics Committee of First Affiliated Hospital of Shantou University Medical College. The patients/participants provided their written informed consent to participate in this study.

## Author Contributions

XT, RL, and YC conceived and designed the study. WO, DL, SN, SC, and ML acquired the data. WO, XH, and JY analyzed the data. YC, WO, and MO'G prepared the manuscript. XT and YC reviewed and edited the manuscript. All authors contributed to the article and approved the submitted version.

## Funding

This work was supported by projects from Grant for Key Disciplinary Project of Clinical Medicine under the High-level University Development Program (2020), Innovation Team Project of Guangdong Universities (2019KCXTD003), Li Ka Shing Foundation Cross-Disciplinary Research Grant (2020LKSFG19B), Funding for Guangdong Medical Leading Talent (2019–2022), National Natural Science Foundation of China (82073659), and Dengfeng Project for the construction of high-level hospitals in Guangdong Province—the First Affiliated Hospital of Shantou University Medical College (202003-2).

## Conflict of Interest

The authors declare that the research was conducted in the absence of any commercial or financial relationships that could be construed as a potential conflict of interest.

## Publisher's Note

All claims expressed in this article are solely those of the authors and do not necessarily represent those of their affiliated organizations, or those of the publisher, the editors and the reviewers. Any product that may be evaluated in this article, or claim that may be made by its manufacturer, is not guaranteed or endorsed by the publisher.

## Abbreviations

BP: blood pressure
HDP: hypertensive disorders of pregnancy
LDH: lactate dehydrogenase
AST/ALT: aspartate aminotransferase to alanine aminotransferase ratio
GGT: gamma-glutamyl transpeptidase
ALP: alkaline phosphatase
UA: uric acid
eGFR: estimated glomerular filtration rate
BMI: body mass index
T: tertile.
