# Supplementary material for: Increased Uric Acid, Gamma-Glutamyl Transpeptidase and Alkaline Phosphatase in Early-Pregnancy Associated With the Development of Gestational Hypertension and Preeclampsia
Source: Front Cardiovasc Med. 2021 Oct 15;8:756140. doi: 10.3389/fcvm.2021.756140 (PMC8554001; doi:10.3389/fcvm.2021.756140)
Supplement: Supplementary file 2 [file Table_2.DOCX]

**Table S2. Longitudinal Associations of Early-pregnancy Biomarkers Levels with Diastolic Blood Pressure Levels during Pregnancy in Women without HDP (*N*=981)**

| Biomarkers | N | Model 1* | Model 2† | Model 3‡ |
| --- | --- | --- | --- | --- |
| UA, µmol/L |  |  |  |  |
| T1 (125−218) | 343 | Reference | Reference | Reference |
| T2 (219−256) | 327 | 0.65 (-0.33, 1.63) | 0.65 (-0.31, 1.60) | 0.62 (-0.34, 1.57) |
| T3 (257−430) | 311 | 2.06 (1.07, 3.06) \|\| | 1.52 (0.54, 2.50) \|\| | 1.19 (0.20, 2.17) § |
| eGFR, mL/min/1.73 m^2^ |  |  |  |  |
| T1 (68−109) | 333 | 1.81 (0.84, 2.79) \|\| | 1.30 (0.27, 2.33) § | 1.17 (0.13, 2.20) § |
| T2 (110−118) | 305 | 1.86 (0.86, 2.86) § | 1.56 (0.53, 2.58) § | 1.43 (0.41, 2.46) § |
| T3 (119−137) | 343 | Reference | Reference | Reference |
| GGT, U/L |  |  |  |  |
| T1 (4−10) | 328 | Reference | Reference | Reference |
| T2 (11−15) | 359 | 1.89 (0.93, 2.86) \|\| | 1.63 (0.68, 2.59) \|\| | 1.42 (0.44, 2.40) § |
| T3 (16−68) | 294 | 2.58 (1.57, 3.59) \|\| | 2.02 (1.02, 3.02) \|\| | 1.36 (0.24, 2.48) § |
| ALP, U/L |  |  |  |  |
| T1 (18−43) | 332 | Reference | Reference | Reference |
| T2 (44−52) | 317 | 1.04 (0.04, 2.03) | 0.91 (-0.05, 1.88) | 0.68 (-0.29, 1.64) |
| T3 (53−193) | 332 | 2.20 (1.21, 3.18) \|\| | 1.53 (0.55, 2.50) § | 1.15 (0.15, 2.14) § |
| LDH, U/L |  |  |  |  |
| T1 (97−139) | 320 | Reference | Reference | Reference |
| T2 (140−157) | 337 | -0.16 (-1.16, 0.83) | 0.01 (-0.96, 0.97) | -0.25 (-1.2, 0.71) |
| T3 (158−273) | 324 | 0.87 (-0.14, 1.87) | 0.66 (-0.31, 1.64) | 0.22 (-0.75, 1.20) |
| AST/ALT |  |  |  |  |
| T1 (0.38−0.97) | 313 | 1.90 (0.90, 2.90) \|\| | 1.08 (0.06, 2.10) § | 0.32 (-0.80, 1.44) |
| T2 (1.00−1.29) | 334 | 1.26 (0.28, 2.24) | 0.80 (-0.18, 1.77) | 0.31 (-0.69, 1.30) |
| T3 (1.30−3.40) | 334 | Reference | Reference | Reference |

Values were based on linear mixed-effects regression models and reflect the difference (95% confidence interval) in maternal diastolic blood pressure for each biomarker level group compared to the reference group. UA, uric acid; eGFR, estimated glomerular filtration rate; GGT, gamma−glutamyl transpeptidase; ALP, alkaline phosphatase; LDH, lactate dehydrogenase; AST/ALT, aspartate aminotransferase to alanine aminotransferase ratio; T, tertile.

*Model 1 was adjusted for gestational age (linear and quadratic terms).

†Model 2 was adjusted for gestational age (linear and quadratic terms), pre-pregnancy BMI, maternal age, monthly per capita income, education level, nulliparous, folic acid supplement intake during pregnancy, family history of hypertension, family history of diabetes mellitus, smoking and alcohol consumption.

‡Model 3 included all six biomarkers simultaneously and was adjusted for gestational age (linear and quadratic terms), pre-pregnancy BMI, maternal age, monthly per capita income, education level, nulliparous, folic acid supplement intake during pregnancy, family history of hypertension, family history of diabetes mellitus, smoking and alcohol consumption.

§*P* <0.05.

|| *P* <0.01.
