# Supplementary material for: Increased Uric Acid, Gamma-Glutamyl Transpeptidase and Alkaline Phosphatase in Early-Pregnancy Associated With the Development of Gestational Hypertension and Preeclampsia
Source: Front Cardiovasc Med. 2021 Oct 15;8:756140. doi: 10.3389/fcvm.2021.756140 (PMC8554001; doi:10.3389/fcvm.2021.756140)
Supplement: Supplementary file 3 [file Table_3.DOCX]

**Supplementary Figure Legends**

**Figure S1.** Scatter plots of maternal systolic blood pressure versus gestational age, according to different early-pregnancy biomarker level groups. The longitudinal trend of systolic blood pressure change with gestational age was predicted by linear mixed-effects regression models. (A) lactate dehydrogenase (LDH); (B) aspartate aminotransferase to alanine aminotransferase ratio (AST/ALT); (C) gamma-glutamyl transpeptidase (GGT); (D) alkaline phosphatase (ALP); (E) uric acid (UA); (F) estimated glomerular filtration rate (eGFR).

**Figure S2.** Scatter plots of maternal diastolic blood pressure versus gestational age, according to different early-pregnancy biomarker level groups. The longitudinal trend of diastolic blood pressure change with gestational age was predicted by linear mixed-effects regression models. (A) lactate dehydrogenase (LDH); (B) aspartate aminotransferase to alanine aminotransferase ratio (AST/ALT); (C) gamma-glutamyl transpeptidase (GGT); (D) alkaline phosphatase (ALP); (E) uric acid (UA); (F) estimated glomerular filtration rate (eGFR).
